# Cytokine Signature in Infective Endocarditis

**DOI:** 10.1371/journal.pone.0133631

**Published:** 2015-07-30

**Authors:** Izabella Rodrigues Araújo, Teresa Cristina Abreu Ferrari, Andréa Teixeira-Carvalho, Ana Carolina Campi-Azevedo, Luan Vieira Rodrigues, Milton Henriques Guimarães Júnior, Thais Lins Souza Barros, Cláudio Léo Gelape, Giovane Rodrigo Sousa, Maria Carmo Pereira Nunes

**Affiliations:** 1 Programa de Pós-Graduação em Infectologia e Medicina Tropical, Departamento de Clínica Médica, Faculdade de Medicina da Universidade Federal de Minas Gerais, Belo Horizonte, Minas Gerais, Brazil; 2 Fundação Oswaldo Cruz, Centro de Pesquisas René Rachou, Laboratório de Biomarcadores de Diagnóstico e Monitoração, Belo Horizonte, Minas Gerais, Brazil; 3 Departamento de Cirurgia, Faculdade de Medicina da Universidade Federal de Minas Gerais, Belo Horizonte, Minas Gerais, Brazil; University of San Francisco, UNITED STATES

## Abstract

Infective endocarditis (IE) is a severe disease with high mortality rate. Cytokines participate in its pathogenesis and may contribute to early diagnosis improving the outcome. This study aimed to evaluate the cytokine profile in IE. Serum concentrations of interleukin (IL)-1β, IL-6, IL-8, IL-10, IL-12 and tumor necrosis factor (TNF)-α were measured by cytometric bead array (CBA) at diagnosis in 81 IE patients, and compared with 34 healthy subjects and 30 patients with non-IE infections, matched to the IE patients by age and gender. Mean age of the IE patients was 47±17 years (range, 15–80 years), and 40 (50%) were male. The IE patients had significantly higher serum concentrations of IL-1β, IL-6, IL-8, IL-10 and TNF-α as compared to the healthy individuals. The median levels of IL-1β, TNF-α and IL-12 were higher in the IE than in the non-IE infections group. TNF-α and IL-12 levels were higher in staphylococcal IE than in the non-staphylococcal IE subgroup. There was a higher proportion of both low IL-10 producers and high producers of IL-1β, TNF-α and IL-12 in the staphylococcal IE than in the non-staphylococcal IE subgroup. This study reinforces a relationship between the expression of proinflammatory cytokines, especially IL-1β, IL-12 and TNF-α, and the pathogenesis of IE. A lower production of IL-10 and impairment in cytokine network may reflect the severity of IE and may be useful for risk stratification.

## Introduction

Infective endocarditis (IE) remains a severe disease associated with a high mortality rate despite recent advances in its diagnosis and treatment [1,2]. Early diagnosis is essential to start effective treatment, especially in staphylococcal IE, which is characterized by a more severe course and a worse outcome [3].

IE is currently diagnosed according to the Duke criteria, a worldwide used system based on clinical features and on echocardiographic and blood culture findings [1,2]. However, the presentation of IE has changed throughout the world, with a reduction in the classic clinical findings traditionally associated with this condition, which can make its diagnosis difficult [1,2,4]. Echocardiography plays a fundamental role in the diagnosis of IE, but it should be interpreted taking into account the clinical features [1,5]. Although blood culture is a valuable tool, the identification of microorganisms on hospital admission has decreased in the past few decades [3,6]. Furthermore, blood cultures pose an inherent delay, which may contribute to the delay in the diagnosis of IE. Therefore, additional diagnostic approach to IE would be of great importance. Considering the infective-inflammatory nature of IE, serum inflammatory markers may contribute to the diagnosis and management of this condition [3,7,8].

Cytokines are small peptides produced when sensitized lymphocytes come directly into contact with an antigen. Their actions are closely connected with the induction and effector phases of all immune and inflammatory responses [9]. Several studies have shown elevation of proinflammatory cytokines in the serum of IE patients compared to health subjects [8,10–13]. However, only in a few studies, the serum concentrations of inflammatory cytokines were compared between patients with IE and controls with non-IE infections [10,14,15].

Therefore, the aim of the present study was to determine serum cytokine concentrations in patients with IE and to compare the levels of these biomarkers with those observed in both healthy individuals and patients with non-IE infections, in order to explore the profile of cytokines in different infectious contexts.

## Materials and Methods

Eighty-one patients with definite IE according to the Duke´s Criteria [1,2] who were admitted to University Hospital, Federal University of Minas Gerais, between January 2005 and May 2013 were enrolled in the study. This institution is a tertiary health care center in the city of Belo Horizonte, Minas Gerais, Brazil. The study was approved by the institution ethics committee and written informed consent was obtained from all participants before sample collection. For patients under 18 years, written informed consent from guardian or parents was also obtained.

Upon admission, the patients underwent a careful clinical examination and a routine laboratory investigation which comprised: blood cultures, complete blood counts, serum C-reactive protein levels, serum chemistry and urine analysis. Transthoracic (TTE) and/or transesophageal echocardiography (TEE) were performed as clinically indicated to investigate: vegetation, abscess, new dehiscence and new moderate or severe valvular regurgitation [16], in addition to preexisting cardiac lesions. Serum samples of all patients were obtained within the first week of admission to measure the concentrations of several cytokines.

In order to compare serum concentrations of the cytokines, two control groups were selected. The first group consisted of 34 healthy blood donors, and the second group comprised 30 patients with non-IE infections. These individuals matched the IE patients by age and gender. Serum samples of them were obtained by the time of their enrollment into the study.

Serum samples from the patients with IE and from the controls were analyzed for the following markers: interleukin (IL)-1β, IL-6, IL-8, IL-10, IL-12 and tumor necrosis factor (TNF)-α. Cytokine concentrations were measured by cytometric bead array (CBA).

### Cytometric bead array assay

The analysis by flow cytometry is a methodology which allows simultaneous measurement of multiple biomarkers in a single sample [17–19]. The CBA immunoassay kit (Becton Dickinson Biosciences Pharmingen, San Diego, CA, USA) was used for the quantitative determination of the cytokines levels according to the instructions of the manufacturer as previously described [17].

The CBA kit comprises 7.5 μm polystyrene microbeads, consisting of six distinct populations, unique on their type-3 fluorescence intensity (FL-3), each coupled to monoclonal antibody (MoAb) against one of the following biomarkers IL-1β, IL-6, IL-8, IL-10, IL-12 and TNF-α (inflammation kit) to capture a given cytokine. A second fluorescently labeled phycoerythrin (PE)-anti-cytokine antibody was added, and the concentrations of the individual cytokines were indicated by their fluorescent intensity. Data were acquired using a FACScalibur flow cytometer (Becton Dickinson, USA). BD FCAP Array software (Becton Dickinson, USA) was used for sample analysis. The results were based on standard concentration curves and expressed as pg/mL.

### Biomarkers networks assembling

Cytokines networks were developed from the correlation of the biomarkers between each other. All the statistically significant correlations (p <0.05) were included in the network and categorized according to the strength of the correlation as follows: low (r <0.35), moderate (r = 0.35 to 0.65) and strong (r >0.65). The significant correlations representing the interactivity between the investigated cytokines were compiled using the open source software, Cytoscape (version 2.8).

### Statistical analysis

Categorical data were presented as numbers and percentages, continuous data were expressed as mean ± standard deviation (SD) or median and interquartile range, as appropriate.

Serum cytokines concentrations were compared using the Kruskal-Wallis test. Post hoc analysis was performed with Dunn´s multiple comparison test. Statistical significance was assumed at p <0.05. The analyses were performed using SPSS statistical software (version 18.0; SPSS, Chicago, IL, USA).

The cytokine profile was assessed to identify low and high cytokine producers. Briefly, after the establishment of the global (IE plus controls) median of the cytokine concentrations, the percentages of individuals showing high cytokine levels, i.e., above the global median value, were calculated for each cytokine. Radars charts were further used to summarize the cytokine signatures. Each axis represents the frequency (%) of cytokine concentrations. The graphics were provided by GraphPad Prism version 5.0 (San Diego, CA, USA).

## Results

### Clinical features

Mean age of IE patients was 47 ± 17 years (range, 15–80 years), and 40 (49%) individuals were male. Characteristics of the study population are shown in Table 1.

**Table 1:** Baseline characteristics of the patients with infective endocarditis.

Variables*	Value
**Rheumatic disease**	32 (39.7)
Intracardiac devices	22 (27.5)
Prosthetic valve	29 (35.8)
Congenital heart disease	12 (15)
Prior infective endocarditis	6 (7.5)
Degenerative valve disease	9 (11.4)
Diabetes mellitus	7 (8.8)
Pharmacologic immune suppression	6 (7.5)
Chronic renal failure	10 (12.5)
Central venous cateter	6 (7.5)
Hepatic insufficiency	3 (3.8)
**Laboratorial data**	
Hemoglobin (g/dl)	10.1 ± 2.0
White blood cell count (x10^3^ cells/l)	13,587.2 ± 6,959.5
C-reactive protein (g/dl)	111± 96.1
**Etiologic agents**	
Streptococci	10 (12.3)
*S*. *aureus*	6 (7.4)
Coagulase-negative staphylococci	10 (12.3)
Enterococci	6 (7.4)
**Echocardiographic findings**	
Chordae rupture	7 (9.0)
Leaflet perforation	21 (26.9)
Abscess	10 (12.3)

*Data presented as mean and standard deviation (mean ± SD) or absolute number (percentage) of patients.

The most common underlying heart disease was rheumatic valvular disease (39.7% of the patients), followed by intracardiac devices such as pacemaker and implantable-cardioverter-defibrillator (21.3%). The mitral valve was the most frequently involved, affecting 34.6% of cases. The portal of entry and source of the bacteremia was identified in 31 patients (38.8%), and dental procedures were the major source of bacteremia (18%).

IE clinical manifestations at presentation included fever (82.3%), anorexia (69.6%), weight loss (65.4%), night sweats (45%), and myalgia (15.8%). Neurological complications were present in 11 patients (13.9%); seven of them were ischemic events. Embolic events also affected other sites, being the lung and kidneys the most frequent ones (three cases each), followed by the spleen (two cases). A systolic murmur was observed in 67 patients (83.3%) on physical examination. The so called classic signs of IE were rarely found and included: Janeway lesions (three cases) and petechiae (five cases). Splenomegaly was observed in 13 patients (16.7%).

#### In-hospital follow-up

Fifty patients (61.6%) developed manifestations of heart failure (HF) during the course of the disease and in 11.4% of them this disorder evolved into refractory HF. Early surgery was performed in 46 (56.8%) cases. HF was the predominant indication for surgery (29.6%), followed by uncontrolled infection (14.8%) and need for device extraction (12.3%). Cardiac complications (perivalvular abscess, chordal rupture, leaflet rupture) were registered in 23 cases (28.6%), and neurological complications occurred in six patients (7.8%). Systemic embolic events were detected in five patients (6.8%), while 20 patients (24.7%) died.

### Laboratorial data

Infective microorganisms were isolated from 38 IE patients (46.8%). The main causative microorganisms were staphylococci (19.7%), streptococci (12.3%), and enterococci (7.4%). Community-acquired methicillin-resistant *Staphylococcus aureus* was found in six patients (7.4%) and coagulase-negative staphylococci in 12.3%. Culture-negative endocarditis occurred in 44 patients (54%) and 25 of them had received antibiotics in the previous month of the diagnosis of IE, with 43% of them in the two weeks prior.

Anemia was present in 86% of the IE patients, and white blood cell count (WBC) was elevated in 65.4%. C-reactive-protein was constantly elevated (mean 111 ± 96 mg/L). Hematuria (32.1%), proteinuria (25.9%) and positive rheumatoid factor (14.8%) were other laboratorial findings at the diagnosis of IE.

Echocardiography was performed in all patients; TTE was performed in 13 subjects (16.5%) and TEE in 68 (83.9%). Vegetations were found in 70 patients (87.3%), with variable length, ranging from 2 mm to 58 mm (median 11 mm; 25 and 75 percentiles: 8 mm and 17.2 mm, respectively). The echocardiographic features of the patients are shown in Table 1.

### Control groups

Thirty-four healthy volunteers were included in the first control group. Mean age was 49.4 ± 18.7 years (range 18–82 years), and 17 (50%) individuals were male. The second control group consisted of 30 patients with active non-IE infections, including pyelonephritis, pneumonia, catheter-related sepsis, and dengue (a tropical virus infectious disease). Mean age of this second control group was 50.4 ± 21.8 years (range 18–85 years), and 16 (53.3%) were male.

There were no significant differences among the three groups regarding age (p = 0.693) and sex distribution (p = 0.933).

### Serum concentrations of the cytokines

**Fig 1:**
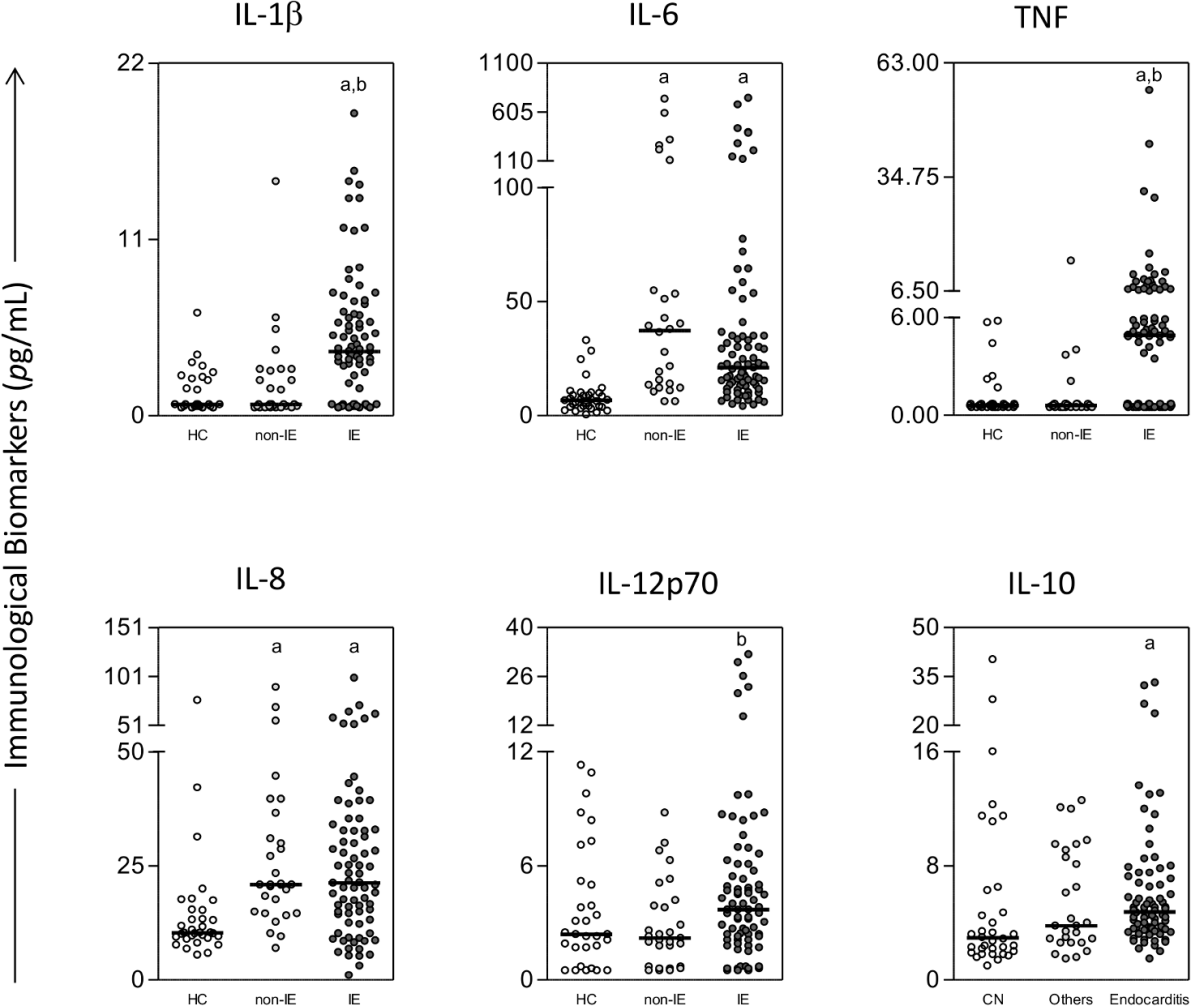
Serum concentrations of inflammatory cytokines in the study groups. The groups evaluated were: health controls (HC) (n = 34, white circle), non-infective endocarditis (non-IE) infections (n = 30, light gray circle), and IE (n = 81, dark gray circle). The results were expressed as pg/mL and a line represented a median value. Significant differences at p<0.05 are highlighted by letters for difference of a group as compared to health controls indicated by ‘‘a” and to non-infective endocarditis indicated by ‘‘b”.

The results of the serum concentrations of the cytokines (IL- 1β, IL-6, IL-8, IL-10, IL-12 and TNF-α) from the patients with IE and from the controls are presented in Fig 1. Except for IL-12, the patients with IE had significantly higher serum concentrations of the cytokines than the healthy donors. Median IL-1β, TNF-α and IL-12 serum levels were significantly higher in the IE patients than in the patients with non-IE infections. When the median serum concentrations of the cytokines were compared between the two control groups, a significant statistical difference was found only for IL-6 and IL-8, which were higher in the group of patients with non-IE infections.

**Fig 2:**
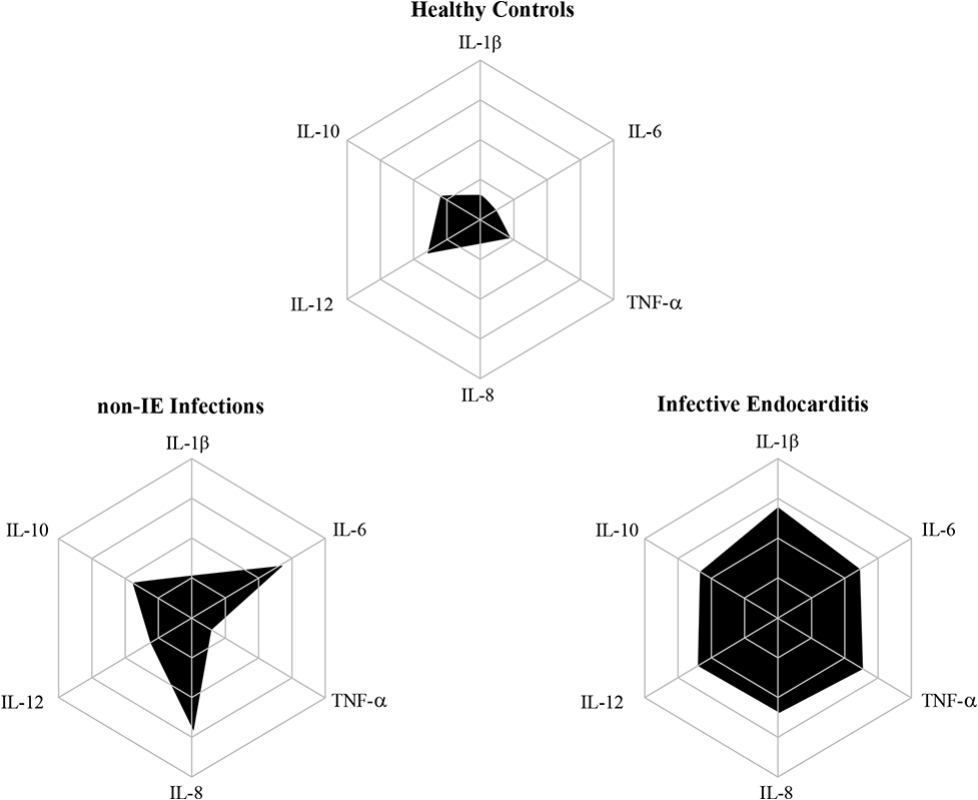
Radar graphics showing the proportion of subjects and the immunological biomarkers production in different contexts. None of the individuals could be identified as high producers of cytokines in the health controls (HC) group. The infective endocarditis (IE) group presented high producers of all cytokines. The non-IE infections group presented high producers of IL-6 and IL-8.

Another way of presenting the results of the serum concentrations of the cytokines is to point out the high producers of these immunological biomarkers in the three study groups. These results are shown in Fig 2. These data complete and illustrate the information given in Fig 1. The IE group included high producers of all cytokines analyzed. The control group of patients with infections other than IE showed an elevated proportion of high producers of IL-6 and IL-8 and a moderate prevalence of high producers of IL-10. When the IE group and the group of other infections were compared, a more elevated proportion of high producers of IL-1β, TNF-α and IL-12 was observed in the IE group. There were high producers only of IL-10 in the group of healthy controls.

**Fig 3:**
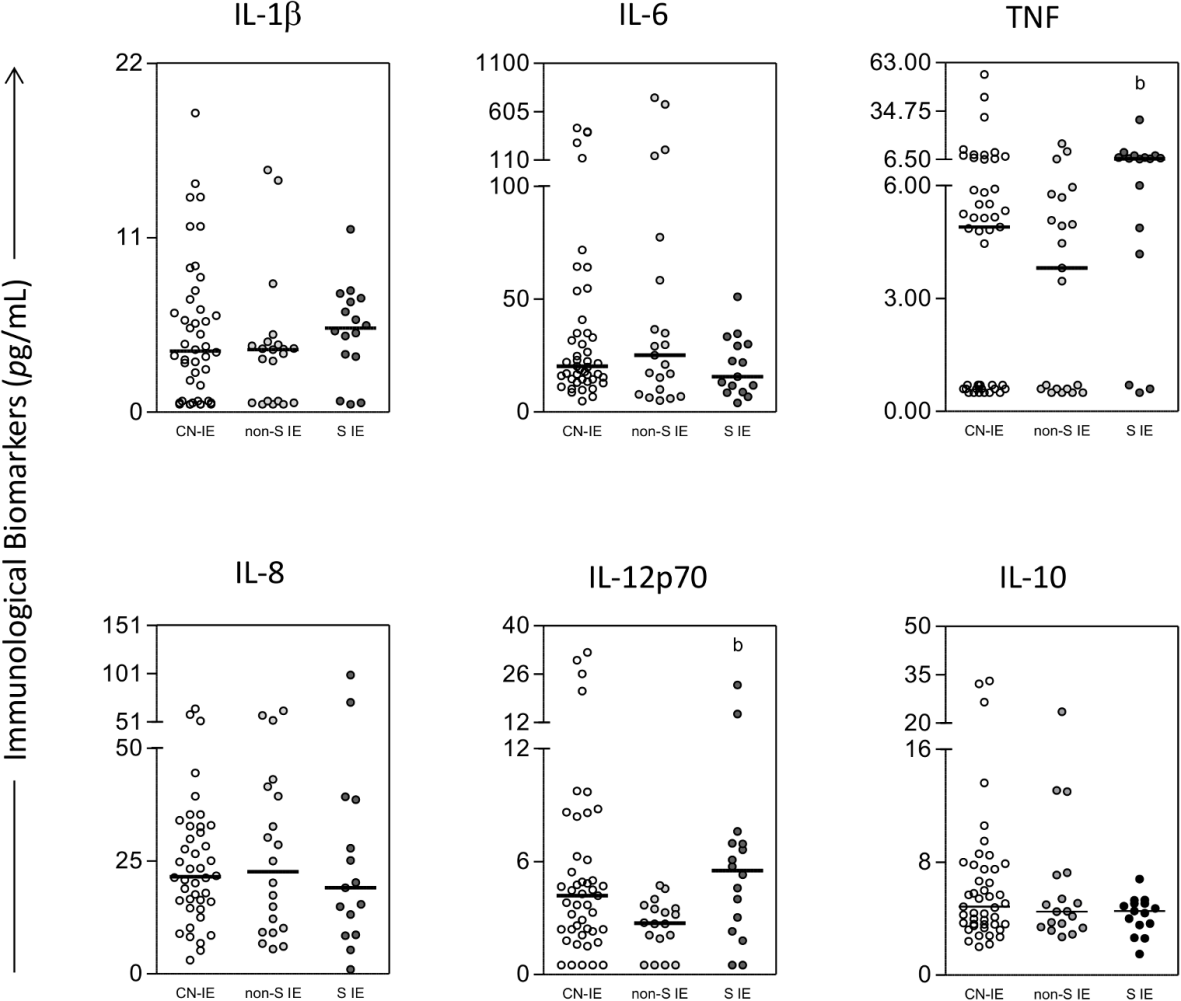
Serum concentrations of inflammatory cytokines in the patients with infective endocarditis (IE) stratified according to the microorganisms. The groups evaluated were: culture-negative IE (CN-IE) (white circle), non-staphylococcal IE (non-S IE) (light gray circle), and staphylococcal IE (S IE) (dark gray circle). Significant differences at p<0.05 are highlighted by letter for difference of a group as compared to non-infective endocarditis indicated by ‘‘b”.

**Fig 4:**
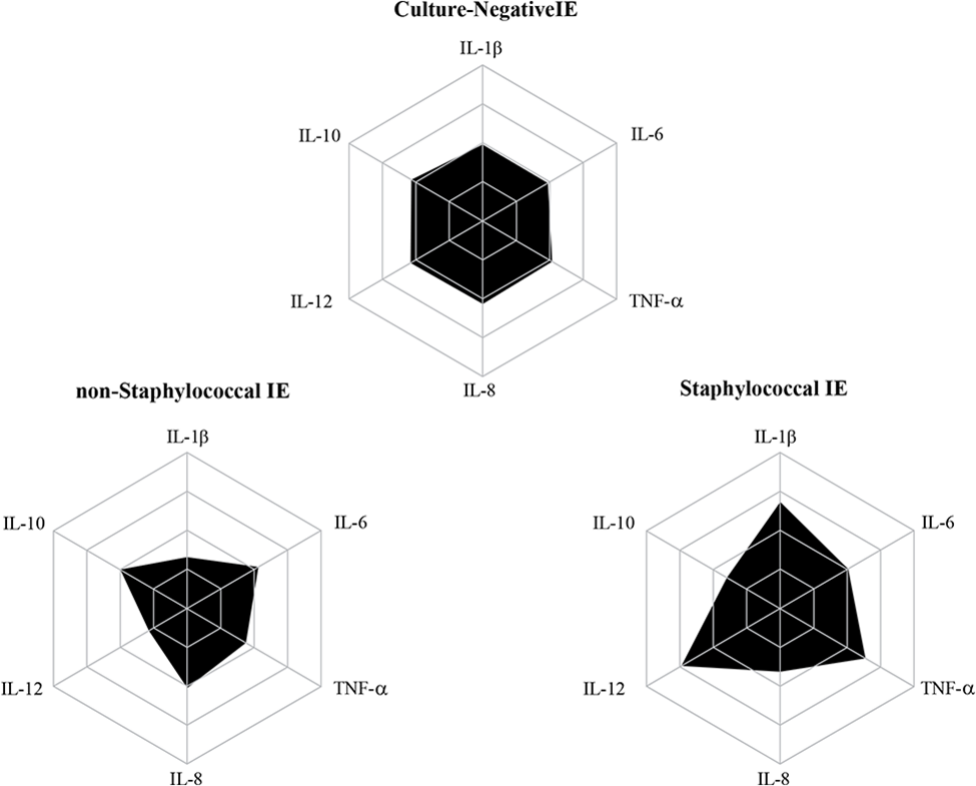
Radar graphics showing the proportion of subjects and the immunological biomarkers production in infective endocarditis (IE) etiological subgroups. The subgroups evaluated were: culture-negative IE (CN-IE), non-staphylococcal IE (non-S IE), and staphylococcal IE (S IE). Elevated proportion of high producers of IL-1β, TNF-α and IL-12 was observed only in the S IE subgroup. The prevalence of high producers of IL-10 was lower in the S IE subgroup as compared to the others subgroups.

In order to assess the levels of the cytokines in the patients of the IE group according to the infecting microorganism, the patients were stratified into three subgroups: staphylococcal IE, non-staphylococcal IE and culture-negative IE. The results are presented in Fig 3. Serum concentrations of TNF-α and IL-12 were significantly higher in the staphylococcal IE subgroup than in the non-staphylococcal IE group. When the prevalence of high producers of the immunological biomarkers among the IE subgroups was investigated (Fig 4), elevated proportion of high producers of IL-1β, TNF-α and IL-12 was observed only in the staphylococcal IE subgroup. In addition, according to Fig 4, the prevalence of high producers of IL-10, an immunoregulatory cytokine, was lower in the staphylococcal IE subgroup. Finally, data from Figs 1 and 3 suggest that there are three distinct clustering of patients: those who present low, medium or high cytokine levels.

**Fig 5:**
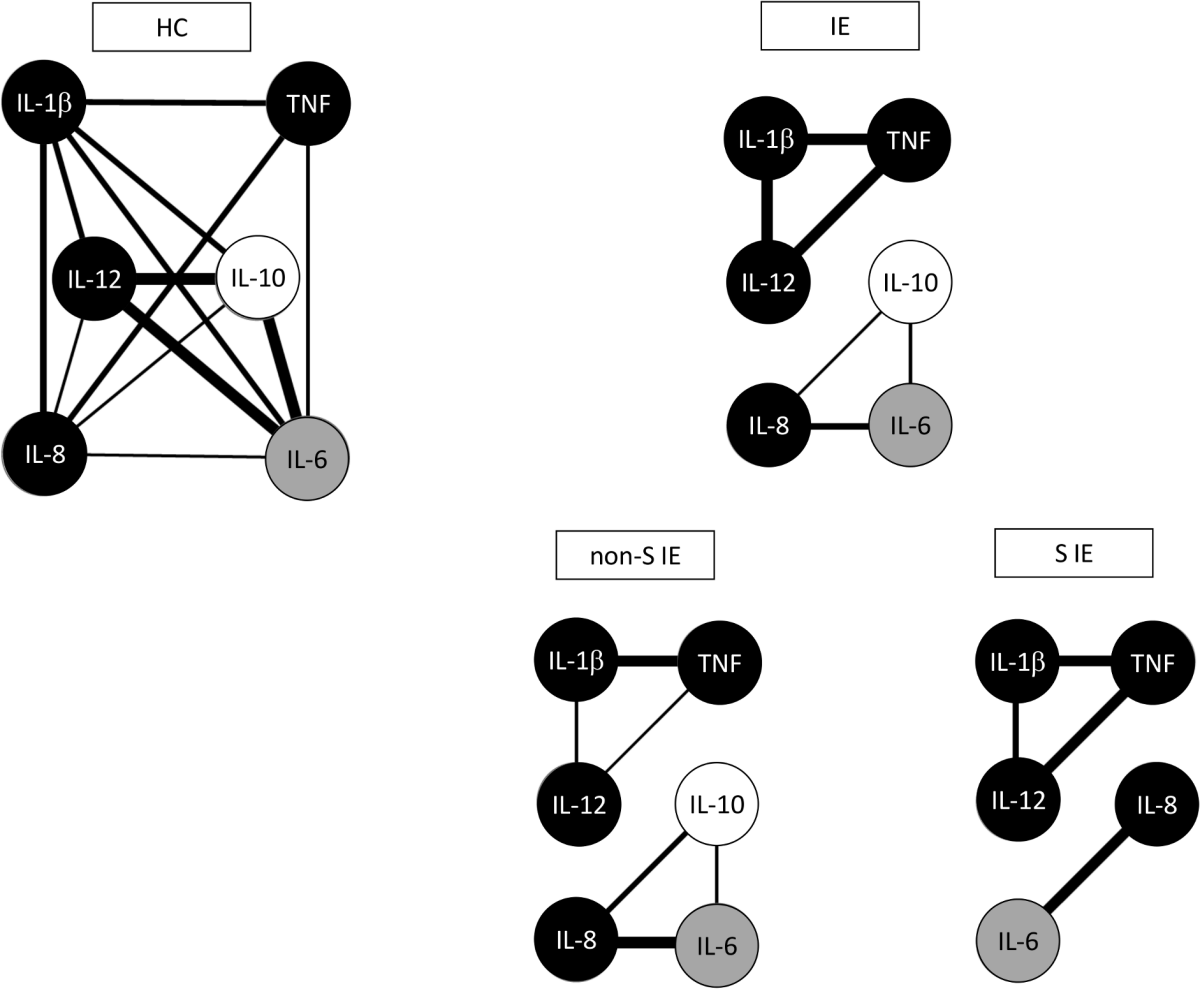
Networks of cytokines associated with Infective endocarditis. The groups evaluated were: health controls (HC), infections endocarditis (IE) and etiological subgroups: non-staphylococcal IE (non-S IE) and staphylococcal IE (S IE). Biomarkers networks were assembled to assess the association between human cytokines for each clinical group. The biomarkers networks were constructed using circle layouts with each cytokines represented by specific gray-scale globular nodes.

Biomarkers networks graphs were constructed to demonstrate the correlation between the pro-inflammatory, regulatory, and balanced cytokines with each other. The network data highlight the loss of interactions between the inflammatory cytokines and IL10 in staphylococcal IE. As a consequence, stronger correlations among the inflammatory cytokines with each other were developed in this subgroup of IE (Fig 5).

### Cytokine levels and outcome

Cytokines levels were compared according to the major symptoms at diagnosis of IE and mortality in Table 2. Patients who presented with fever had more elevated concentration of the cytokines than those without this symptom. Additionally, IL-8 was higher in the patients who died in comparison with those who remained alive, whereas IL-6 and IL-10 tended to be lower in the individuals who survived (Table 2).

**Table 2:** Comparison between cytokine levels and major symptoms at diagnosis of infective endocarditis and mortality.

	Symptoms		
Cytokines*	Present	Absent	P-value
	**Fever**		
Interleukin-1β	3.7 (1.8–6.7)	0 (0–4.4)	0.026
Interleukin-6	22.6 (13.3–46.1)	15.5 (10.8–22.8)	0.071
Interleukin-10	4.9 (3.7–7.7)	3.7 (2.6–5.8)	0.050
Interleukin-12	3.7 (2.1-.0)	1.4 (0–3.6)	0.005
TNF-α	4.6 (0–6.7)	0 (0–4.6)	0.038
	**Sweats**		
Interleukin-1β	3.8 (2.7–7.2)	3.0 (0–5.2)	0.088
Interleukin-12	4.0 (2.3–7.1)	2.6 (1.3–4.3)	0.028
TNF-α	4.8 (3.7–6.5)	0 (0–5.4)	0.014
	**Anemia**		
Interleukin-6	22.3 (14.6–52.9)	13.2 (6.8–17.9)	0.011
Interleukin -8	25.1 (15.4–39.3)	14.9 (5.2–19.1)	0.010
Interleukin-10	5.0 (3.7–7.7)	3.2 (2.7–5.6)	0.024
	Leukocytosis		
Interleukin -8	28.2 (16.2–41.9)	17.4 (8.8–26.4)	0.003
	**Heart failure**		
Interleukin -1β	3.8 (3.1–6.7)	3.2 (0–5.6)	0.048
Interleukin-12	5.1 (2.2–7.3)	2.8 (1.4–4.3)	0.031
	**Death**		
Interleukin -6	31.3 (17.1–147.5)	18.8 (13.0–33.8)	0.057
Interleukin-8	32.1 (20.5–58.5)	20.2 (10.8–32.7)	0.032
Interleukin-10	5.9 (4.4–11.2)	4.6 (3.5–5.9)	0.056

TNF-α, tumor necrosis factor-α.

*Median (interquartile range), pg/mL.

## Discussion

In this study, we evaluated the profile of cytokines in IE, non-IE infections and healthy individuals. Our findings showed that the patients with IE presented significantly higher serum concentrations of IL-1β, TNF-α and IL-12 than the matched-controls with non-IE infections. Additionally, the patients with IE had higher serum concentrations of the immunological biomarkers than the healthy volunteers, except for IL-12. When the IE patients were stratified according to the infecting microorganism, we found higher serum concentrations of TNF-α and IL-12 in staphylococcal IE than in non-staphylococcal IE patients. Furthermore, elevated prevalence of high producers of IL-1β, TNF-α and IL-12 was observed only in the staphylococcal subgroup.

Early diagnosis of IE is important for improving the clinical outcome as late diagnosis is related to increased mortality. The diagnosis of this condition is based on clinical features, and echocardiographic and blood culture findings. The clinical profile of IE patients has changed in the last decades [2,20], and the percentage of negative blood cultures has significantly increased [3,6]. Although echocardiography is of fundamental importance in the diagnosis of IE, its sensitivity is lower in some contexts especially in IE related to intracardiac devices, preexisting severe lesions, very small vegetations, and non-vegetant IE [1]. Therefore, the importance of additional criteria for diagnosing IE is evident. Measurement of inflammatory cytokines could contribute to the diagnosis of this disorder.

The term cytokine describes a functional class of small protein mediators which participates in the induction and effector phases of all immune and inflammatory responses [9]. Once released, proinflammatory cytokines activate the innate and/or adaptive immune response, which lead to further production of immunoregulatory and/or effector cytokines [21]. Several cytokines are essential for initiating and maintaining the inflammatory response by recruiting leukocytes to the sites of infection [3,4,7,10,22].

Previous studies support that proinflammatory cytokines are elevated in the serum of IE patients compared to healthy individuals [8,10,11,13,23], which was confirmed in the present study. However, we observed similar IL-12 levels between IE patients and the healthy controls. IL-12 participates in the regulation of innate and adaptive immune response [24], and may have a central role in the development and progression of cell-mediated autoimmune diseases, as well as in the maintenance of inflammation in atherosclerotic disease [25]. Gram-positive bacteria, such as streptococci and *S*. *aureus* interact with Toll like receptor (TLR) type 2, which is expressed in different cell populations of the innate immunity, including dendritic cells. After this interaction, the dendritic cells secrete IL-12, which is important to induce CD4^+^ T cells to differentiate into Th1 cells that drive the inflammatory immune response [26]. The polymorphism of TLR2 [27] and TLR4 [28] has been described to increase the risk of developing IE, which may be due to low secretion of IL-12. This phenomenon could have contributed to the lack of difference of the levels of this cytokine between the healthy individuals and the patients with IE. TLR polymorphism could also have occurred in the healthy controls causing difficulty in the understanding of the findings related to IL-12 in the present study. Furthermore, although patients with active infections or autoimmune diseases were not included among the healthy individuals, the existence of undiagnosed disorders cannot be completely ruled out. The mean age of the healthy volunteers was higher in comparison with the other groups; and asymptomatic chronic diseases might be present. These facts could also explain why only serum concentrations of IL-6 and IL-8 were significantly higher in the patients with non-IE infections when this group was compared to the healthy controls.

Differently from our study, Kern *et al*. [10] found low or undetectable serum levels of TNF-α in the sera of patients with IE. Similarly, Rawczynska-Englert *et al*. [14] observed undetectable serum levels of IL-1α. These results might be explained by different kinetics of particular cytokines, such as renal clearance and half-life in addition to total amount [14]. Furthermore, the differences in serum cytokines detection observed between these studies and ours may be due, at least partially, to the higher sensitivity of the technique employed in our investigation.

Only in a few studies, serum concentrations of cytokines from IE patients were compared to those from individuals with non-IE infections [10,14,15]. Rawczynska-Englert *et al*. [14] measured the levels of IL-1α, IL-6 and TNF-α in the serum from 40 patients with acquired rheumatic valvular heart disease and IE. The serum concentrations of the same cytokines were obtained from two control groups comprised of patients with acquired rheumatic valvular heart disease: 15 without any infection and 15 with active urinary tract infection (UTI). In all controls without infection, IL-6 concentrations were below the calibration range. In the other two groups, this cytokine was detected, with higher levels in the IE group when compared to the controls with UTI. Although the difference of IL-6 concentrations between these two groups was not statistically significant, the authors concluded that serum IL-6 levels might suggest ongoing IE and might be used to aid in diagnosis of the disease.

In our study, we included a larger matched-control with active non-IE infections with the purpose of simulating the diagnostic challenge that often occurs in clinical practice. We observed that the median IL-1β, IL-12 and TNF-α serum levels were significantly higher in IE than non-IE infections patients.

The pathophysiology of IE is closely related to the inflammatory response. Inflamed endothelia produce cytokines, integrins and tissue factor which, in turn, attract fibronectin, monocytes, and platelets. Bacteria attaching to such structures further activate the inflammatory cascade [5]. Therefore, the high levels of potent proinflammatory cytokines, such as IL-1β and TNF-α are not surprising. Previous studies suggest that TNF-α coordinates the cytokine response to injury and acts as a fire alarm [21,29]. Likewise, IL-1β is a pivotal mediator for the development of the acute phase response [21,30].

### Serum cytokines levels in staphylococcal IE

When the IE group was stratified according to the etiologic agent, high producers of IL-1β, IL-12 and TNF-α was observed only in staphylococcal IE subgroup. Similarly, Nunes *et al*. [8] verified that IL-12 and IL-1β serum concentrations were higher in patients with IE caused by *S*. *aureus* compared with streptococcal IE. These findings are in agreement with the observation that pathogens can differ in their abilities to induce inflammatory and immunoregulatory cytokines [7]. In contrast to streptococci, *S*. *aureus* is able to invade intact endothelial cells in addition to colonize damaged endothelium [5]. It is well known that *S*. *aureus* is associated with both a higher rate of complications and mortality in IE [3,5,13].

Considering the virulent nature of *S*. *aureus*, higher serum concentrations of IL-1β and TNF-α in staphylococcal IE are not surprising since an exacerbated inflammatory response was expected and those are potent inflammatory cytokines. Higher serum concentrations of IL-12 are also in agreement with the pathogenesis of staphylococcal IE. IL-12 regulates the innate immune response and promotes the development of a T helper 1 (Th1) adaptive immune response, which is characterized by enhanced mononuclear phagocyte activities [21,24].

In addition, according to our findings (Figs 4 and 5), the inflammatory response in staphylococcal IE may not be properly antagonized by IL-10 production. Inflammatory response successfully eradicates pathogens, but often causes deleterious side effects. IL-10 has anti-inflammatory effects, which regulate the immune response, limiting damage to the host and preventing immunopathology [31,32]. We hypothesize that the lower production of IL-10 in staphylococcal IE could explain, at least in part, the more exacerbated inflammatory response and the higher clinical impact of this infection. However, it remains uncertain whether initial high IL-12, IL-1β and TNF-α levels and/or low IL-10 concentrations are associated (or not) with a worse prognosis.

In summary, data analysis demonstrated that the staphylococcal IE patients presented significantly higher levels of TNF-α and IL-12 than the other subgroups. This result lead us to speculate that these cytokines may be involved in the increase of vascular permeability, migration of lymphocytes to the inflammation sites as well as IFN-*γ* induction of inflammation mediated by macrophages and lymphocytes, causing a highly inflammatory response in *Staphylococcus spp*. IE. However, further studies are necessary to clarify the role of these cytokines in the exacerbated inflammation observed in staphylococcal IE. In addition, there was a trend of inflammation mediated by IL-6 in the non-staphylococcal IE when comparing with IE caused by staphylococci. Another observed trend was the higher levels of IL-1β in culture negative IE in comparison with the other two subgroups.

### Network analysis

The evaluation of cytokine levels allowed the development of networks correlating the cytokines with each other in the studied groups. The connections among the cytokines in the healthy group showed strong connections among IL-12, IL-10 and IL-6 suggesting that these cytokines are necessary to maintain health, regulate and avoid inflammation. On the other hand, in the IE group we verified strong connections among TNF-α, IL-1β and IL-12, which were highly expressed and, therefore, are probably related to the exacerbated inflammation observed in IE. It should be highlighted the fragile connection between IL-8 and IL-10 in the healthy controls and IE patients, which suggests that these cytokines may be important determinants of the threshold between regulation and inflammation. In the context of Staphylococcal IE, there were strong connections between IL-1β and TNF-α and also between TNF-α and IL-12, which is in agreement with the high inflammatory profile induced by *Staphylococcus spp*. We ascribed the stronger connections between the inflammatory cytokines in this subgroup to the absence of IL-10, which presents important anti-inflammatory effects.

### Study limitations

Our study group included patients from a large tertiary-care center, to which many patients with IE were referred from other institutions. This fact may have caused selection bias which limits the generalization of the results. Furthermore, as previously mentioned, although efforts were made in order to select completely healthy individuals, the existence of undiagnosed and asymptomatic disorders cannot be completely ruled out.

In conclusion, the findings of this study reinforce a relationship between the expression of proinflammatory cytokines, especially IL-1β, IL-12 and TNF-α, and the pathogenesis of IE. The more exacerbated inflammatory response and the higher severity of the staphylococcal IE as compared to the non-staphylococcal ones might be associated, at least in part, with a lower production of IL-10. Impairment in the cytokine network may be an important factor associated with IE morbidity. Finally, immune process in IE is very complex, and multiple mechanisms may be involved. Further studies are necessary to define the role of inflammatory cytokines in the diagnosis and prognosis of IE.
